# α-ketoglutarate ameliorates colitis through modulation of inflammation, ER stress, and apoptosis

**DOI:** 10.1016/j.toxrep.2025.101897

**Published:** 2025-01-06

**Authors:** Ankita Mandal, Sharmistha Banerjee, Sumit Ghosh, Sima Biswas, Angshuman Bagchi, Parames C. Sil

**Affiliations:** a Division of Molecular Medicine, Bose Institute, P-1/12, CIT Scheme VII M, Kolkata, West Bengal 700054, India; b Department of Biochemistry and Biophysics, University of Kalyani, Nadia, Kalyani, West Bengal 741235, India

**Keywords:** Colitis, α-ketoglutarate, TNBS, Oxidative stress, Inflammation, ER stress, Apoptosis

## Abstract

Colitis is an inflammatory disorder of the gastrointestinal tract. A widely consumed dietary nutrient, α-ketoglutarate (α-KG) is known to play a crucial role in cellular metabolism and provide protection to intestinal epithelium under various pathophysiological conditions. In this study, 2,4,6-trinitrobenzenesulfonic acid (TNBS) was used to induce colitis in Wistar rats. After 36 hours of TNBS administration, the rats were orally treated with a solution of α-KG at 1 g/kg body weight for 5 days. Development of colitis was confirmed by observable physical symptoms of repeated loose blood-mixed stool, apathy for food and weight loss. Macroscopic inspection revealed an inflamed colonic surface with ulcerations. Histopathological observations included alterations in crypts-structure and disruption in both epithelial and mucosal layers of colon in colitis induced rats. Colitis resulted in elevated levels of pro-inflammatory cytokines, ER stress-mediated cell death and intrinsic apoptosis pathway. The ameliorative effects of α-KG against TNBS-mediated toxicity were confirmed through molecular technics and docking analysis. Additionally, there were no instances of toxicity of α-KG. Therefore, α-KG can be considered as a valuable therapeutic agent for further comprehensive research.

## Highlights

• α-KG improves colitis-induced pathophysiological and histopathological alterations.
• α-KG ameliorates inflammation, ROS and ER stress-induced apoptosis in colitis.
• α-KG has the highest binding affinity for NF-κB complex than IKK and TNF-R1.

## Graphical Abstract

**Figure:**
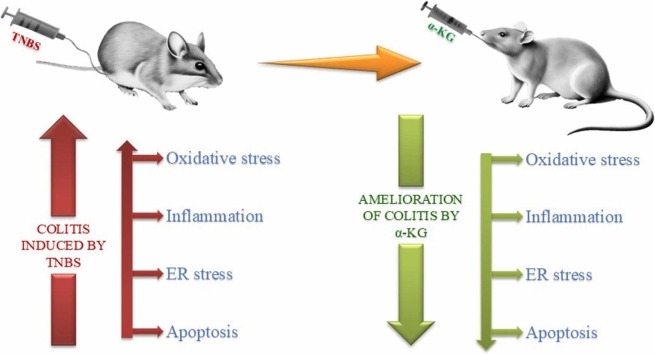

## 1 Introduction

Colitis is an important feature of inflammatory bowel disease (IBD), where chronic inflammation of idiopathic origin is seen in the gastrointestinal (GI) tract. Colitis and Crohn’s diseases represent two major forms of IBD. Onset of colitis is characterized by various symptoms like frequent diarrhea, loose stool without or with blood, apathy for food accompanied by weight loss [1], [2]. Developed nations such as USA, Australia and several European countries show high prevalence of IBD [3], [4]. Apart from these regions, the number of IBD patients is increasing in developing countries as well [5]. Recent reports indicate that, although the incidence rate in high-income countries remains relatively stable, it is escalating in low and middle-income countries, predominantly affecting middle aged individuals. As a result, the global burden of colitis is significantly expanding, with an estimation of total 5 million cases approximately and addition of 156–290 new cases per 100000 individuals annually [6].

The exact cause of colitis is still unidentified and multiple factors are assumed to attribute as its etiopathology. Genetic and environmental factors, along with microbial dysbiosis and dysregulated immune responses are key players for the beginning of this illness [7], [8]. In the pathogenesis of colitis, pro-inflammatory cytokines like TNF-α, IL-1β and IL-18 are the chief contributing factors [9], [10]. Increased oxidative stress paired with decreased levels of antioxidants are common occurrences in colitis [11], [12]. Additionally, colitis is closely associated with ER stress and apoptosis [13], [14].

Despite better comprehension and advanced medications with anti-inflammatory drugs, immune-suppressors and biologics, significant percentage of patients still experience treatment failure. In such instances, surgical intervention is the ultimate resort. Unfortunately, such management approach leads to high percentage of morbidity and is financially burdensome too [15]. Therefore, it is imperative to seek a reliable stable agent which is readily accessible, easy to administer with minimal side effects and cost-effective as well [6].

Recent research on nutrients has highlighted that α-KG, a metabolic intermediate in the TCA cycle, not only improves the nutritional status but also modulates various metabolic processes. Diverse bioactive actions of α-KG reinforce the intestinal epithelium to maintain gut homeostasis [16]. The production of α-KG is facilitated by two pathways: decarboxylation of isocitrate catalyzed by isocitrate dehydrogenase and oxidative deamination of glutamate by glutamate dehydrogenase [17]. Since the α-KG generated by TCA cycle cannot be directly utilized in human body, it is essential to offer dietary supplementation of α-KG to ensure a consistent supply [18]. Studies have shown that dietary α-KG supplementation raised its intracellular concentration which inhibited NF-κB mediated inflammatory pathway [19]. α-KG generates plenty of ATP via TCA cycle and caters to the energy requirement of rapidly dividing intestinal epithelial cells. Furthermore, it protects the intestinal mucosal layer by its antioxidant capability [20]. Recent research demonstrated that α-KG improves the level of SOD, GSH and catalase (CAT), highlighting its antioxidative property [21]. α-KG neutralizes ROS by enhancing the activity of antioxidant enzymes as well as scavenges ROS directly by oxidative decarboxylation of H_2_O_2_ [22]. It also prevents apoptosis by elevating the expression of nuclear factor, Nrf2 [23], [24].

In recent years, extensive investigations have been conducted regarding protective effects of α-KG. However, beneficial role of α-KG in colitis-related pathophysiology remains largely unexplored. Therefore, in this study, our objective was to investigate the therapeutic efficacy of α-KG in alleviating colitis-associated pathophysiology.

## 2 Materials and methods

### 2.1 Chemicals

TNBS, α-KG, protease and phosphatase inhibitor cocktails were bought from Sigma aldrich (USA). EDTA, BSA and MgCl_2_ were bought from SRL, India. Glycerol and formalin were acquired from Merck (Germany). All the antibodies utilized in this research were acquired from Cell signaling technology Inc. (USA), Abcam (UK) and Novus biologicals (USA).

### 2.2 Animals

Male Wistar rats aged around 4 weeks and weighed approximately 150–200 g were selected and primarily acclimatized under standard laboratory environment for 2 weeks. The animals were provided with conventional diet and exposed to 12 hours cycles of light & darkness. All the experiments were performed following the guidelines of the Institutional Animal Ethics Committee (IAEC), Bose Institute, Kolkata, India (IAEC/BI/139/2019). Approval for conducting investigations was obtained by both IAEC and The Committee for The Purpose of Control and Supervision on Experiments on Animals (CPCSEA), Ministry of Environment and Forests, New Delhi, India (1796/PO/Ere/S/14/CPCSEA).

### 2.3 Experimental procedure

Induction of colitis was carried out following the protocol established by Wirtz et al. [25]. At first, a solution of 1 % (w/v) TNBS was prepared by combining 4 volumes of acetone & olive oil mix (in the ratio of 4:1) with 1 vol of 5 % H_2_O-TNBS solution and applied on the back surface on the earlobes of rats for pre-sensitization. After application, rats were closely monitored for 7 days. For colitis induction, rats were anesthetized on the 8th day and 500 µl TNBS solution [5 % (w/v) H_2_O-TNBS solution with ethanol in a 1:1 ratio] was administered intra-rectally by catheter insertion. The progression of colitis induction was assessed through the analysis of biological and pathophysiological symptoms in TNBS induced rats, which included calculation of overall body weight loss and observation of excretion of soft & bloody stool. The severity of colitis was assessed using a Disease Activity Index (DAI) (Table 1). Rats were monitored daily and DAI scores were documented to evaluate the treatment's effectiveness. The animals were euthanized through cervical dislocation after the whole treatment procedure mentioned below and the colons were dissected out & cleansed to observe the colonic mucosal surface with ulcerations.

**Table 1:** Disease activity index (DAI) score.

**Score**	**Weight Loss**	**Stool consistency**	**Bleeding**	**Activity/behaviour**
**0**	None	Normal	No bleeding	Normal
**1**	1–5 %	Loose stools	No bleeding	Slightly reduced activity
**2**	5–10 %	Loose stools	Slight hemoccult	Mild reduction in activity
**3**	10–15 %	Slight diarrhea	Severe hemoccult	Moderate reduction
**4**	More than 15 %	Watery diarrhea	Gross bleeding	Lethargy

#### 2.3.1 Basic study design

**Fig. 1:**
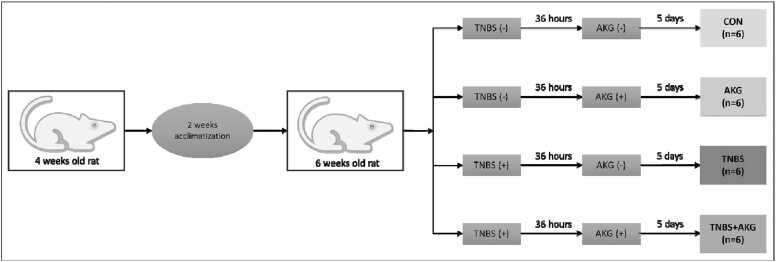
*In vivo* experimental design. CON: animals were solely provided with a standard diet, AKG: experimental animals administered with α-KG dissolved in sterile water, TNBS: TNBS administered intrarectally for colitis induction, TNBS+AKG: α-KG administered orally 36 hours after TNBS administration and continued once daily for 5 days.

Animals were separated into 4 groups comprising of 6 rats in each group (Fig. 1).

Group 1 (CON): Control group. Animals received a normal diet only.

Group 2 (AKG): Rats were orally administered a solution containing α-KG dissolved in sterile water with the help of gavage needle, at a dosage of 1 g/kg body weight, once daily for 5 consecutive days.

Group 3 (TNBS): TNBS was administered in rats for colitis induction following above-mentioned procedure.

Group 4 (TNBS+AKG): After 36 hours of TNBS administration, rats were orally provided with a solution of α-KG dissolved in sterile water at a dose of 1 g/kg body weight by gavage needle. Administration of α-KG was continued for additional 4 days.

The specified dosage of α-KG was determined by consulting the studies conducted by Bhattacharya et al. [26], [27], [28].

Upon completion of the entire treatment procedure, the animals were euthanized by cervical dislocation; the colons were dissected, cleaned with 1X PBS and stored at −80^ᵒ^ C for further experimental purposes.

### 2.4 Study of histological sections

The colon tissues were fixed in buffer solution of 10 % formalin for 24 hours at room temperature. Then they were subjected to paraffin embedding. Thin section of tissues, obtained through microtomy, was subjected to hematoxylin and eosin staining. The alterations in the architecture and morphology of colon tissue were visually analyzed under bright field microscope (Leica 269 Microsystem DN1000 with camera: DFC450C). A histological grading system was used to evaluate the disease progression and determine the effects of α-KG on the colitis (Table 2).

**Table 2:** Histological grading of colitis.

**Grading Features**	**Grade**	**Description**
Inflammation	0	None
	1	Slight
	2	Moderate
	3	Severe
Extent	0	None
	1	Mucosa
	2	Mucosa and submucosa
	3	Transmural
Regeneration	0	Complete regeneration or normal tissue
	1	Almost complete regeneration
	2	Regeneration with crypt depletion
	3	Surface epithelium not intact
	4	No tissue repair
Crypt damage	0	None
	1	Basal 1/3 damaged
	2	Basal 2/3 damaged
	3	Only surface epithelium intact
	4	Entire crypt and epithelium lost
Percent involvement	0	No % involved
	1	1–25 % involved
	2	26–50 % involved
	3	51–75 % involved
	4	76–100 % involved

### 2.5 Study of MPO activity

MPO activity was assessed following the methodology outlined by Bradley et al. The rate of H_2_O_2_-dependent oxidation of dianisidine catalyzed by MPO was measured spectrophotometrically at an absorbance of 460 nm [29].

### 2.6 Measurement of GSH

Intracellular GSH content of resected colon tissue was quantified by the method described by Bhor et al. Colon tissue homogenates were first precipitated using 5 % TCA reagent. After removal of the protein precipitate, DTNB (known as Ellman’s reagent) was added. Absorbance was recorded at 412 nm [30], [31], [32].

### 2.7 Measurement of catalase activity

Catalase activity of experimental rat colons was determined by tracking the decomposition of H₂O₂ for 10 minutes, following the method described by Cohen et al. The decomposition was monitored spectrophotometrically at 240 nm [33], [34].

### 2.8 Extraction of RNA and reverse transcriptase-PCR

Colon tissue resection followed by RNA extraction was performed using TRIZOL reagent, following the manufacturer’s instruction (Invitrogen, Carlsbad, CA). Post isolation, RNA was quantified utilizing nanodrop spectrophotometer with a Hellma TrayCell Type 105.810 (Hellma Analytical). cDNA was yielded from 2 µg amount of RNA using the Thermo Scientific Verso cDNA synthesis kit (Thermo Scientific, USA), from each experimental group. Thermal cycling was executed in the following manner: (95^°^ C, 5 minutes) followed by 35 cycles of: (95^°^ C, 30 seconds; T_m_^°^ C, 30 seconds and 72^°^ C, 45 seconds). The step of DNA extension was performed at 72^°^ C for 5 minutes. Then the obtained PCR products were stored at 4^°^ C, followed by agarose gel electrophoresis using a 1.5 % agarose gel. The primer credentials are provided in Table 3 [30], [35], [36].

**Table 3:** List of primers used in this study.

**Gene**	**Primer Sequence** **(5’-3’)**	**Annealing Temperature (**^**o**^**C)**	**Amplicon Size** **(bp)**
IL−6	*Fp: CAGAGCAATACTGAAACCCTAGT* *Rp: TTCTGACCACAGTGAGGAATG*	50.5	262
IL−1β	*Fp: CTTCCTAAAGATGGCTGCACTA* *Rp: ATCCCATACACACGGACAAC*	50.1	307
COX−2	*Fp: GAACTTAGGCCATTGGAATTTACT* *Rp: CAGTTCTCGATGCACAAATTCT*	55.1	192
MCP−1	*Fp: GTGTCCCAAAGAAGCTGTAGTA* *Rp: AAGGCATCACATTCCAAATCAC*	50.5	297
ICAM−1	*Fp: CACCATGCTTCCTCTGACAT* *Rp: CACTGCTCGTCCACATAGTATT*	50.5	283
VCAM−1	*Fp: GAGTGCAAGAAGCCAACTAGA* *Rp: AGCTGCCTACTCAACATTAACA*	50.5	258
TNF-α	*Fp: CTGAAGTAGTGGCCTGGATTG* *Rp: GCTGGTAGTTTAGCTCCGTTT*	50.5	424
β-Actin	*Fp: TCCCTGGAGAAGAGCTATGA* *Rp: ATAGAGCCACCAATCCACAC*	50.7	332

### 2.9 Immunoblotting

Protein was isolated from colon homogenates and equal quantity of protein (50 µg) from respective experimental groups were then resolved through SDS-PAGE (10–20 %) and transferred to the PVDF membrane [37]. The membranes were then individually incubated with anti-β-actin, anti-caspase 12, anti-caspase 3, anti-calpain 1 and anti-GRP78 antibodies with the recommended dilution of 1:1000, at 4^°^ C overnight. The membranes were cleansed with TBST and allowed to react with the HRP-conjugated secondary antibody for 30 minutes (dilution ratio of 1:20,000). The levels of protein expression were studied using ECL solution [38], [39].

### 2.10 Molecular Docking simulation

The crystal structures of inhibitor of nuclear factor-κB (IκB) kinase (IKK), NF-κB and TNFR-1 were collected from the Protein Data Bank (PDB) (https://www.rcsb.org/) bearing the PDB IDs 3BRV, 1NFI & 1EXT respectively. For the purpose of finding the binding interactions between the ligand α-KG and aforementioned protein receptors, we extracted the structure of α-KG from PubChem database (PubChem ID:78866) then the α-KG structure was converted in DS 2.5 server. The structures of the receptors and the ligand were optimized by removing the unwanted steric clashes from the structures by the process of energy minimizations in the Discovery Studio 2.5 (DS 2.5) platform. The ligand was prepared using DS 2.5 servers prepare ligand protocol. Furthermore, the heteroatom records were removed from the structures of the receptor proteins. Then the process of molecular docking simulations was performed between the receptors and the ligand with the help of the tool AutoDock 4.2 [40]. Each docking simulation produced 10 docking poses and out of them the best pose was selected on the basis of the binding free energy values of the complexes. The selected poses were subsequently analysed in the DS 2.5 platform and the modes of interactions between the receptors and ligand were elucidated [41].

### 2.11 Statistical analyses

All values have been conveyed as mean ± SEM for 3 independent experiments. One-way ANOVA was performed and comparison among the group means were accomplished by using the Tukey test. Origin8 software (OriginLab, Massachusetts) was used for statistical calculations. Rendered p- value for statistical significance was less than or equal to 0.05.

## 3 Results

### 3.1 Effect of α-KG on TNBS induced colitis

**Fig. 2:**
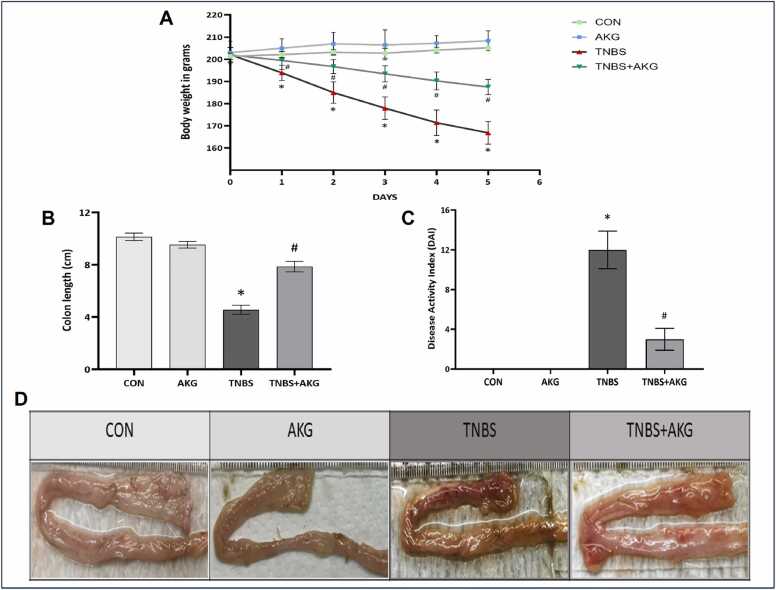
Effect of α-KG on TNBS mediated colitis induction. (A) Change in body weight during progression of colitis. (B) Length of colon in different experimental groups. (C) Disease activity index of animals from each group. (D) Depiction of macroscopic picture of colon tissue. CON: animals were solely provided with a standard diet, AKG: experimental animals administered with α-KG dissolved in sterile water, TNBS: TNBS administered intrarectally for colitis induction, TNBS+AKG: α-KG administered orally 36 hours after TNBS administration and continued once every day for 5 days. The final results have been expressed as mean ± SEM of 3 independent experiments of each group. *P < 0.05 vs CON; #P < 0.05 vs TNBS.

We examined the effects of α-KG on colitis by assessing changes in body weight, colon length, and DAI. Decrease in body weight, coupled with shortening of colon length, and colonic ulceration with presence of fecal blood were observed in diseased rats. Treatment with α-KG showed recovery in body weight, less frequent passage of formed stool with less amount of blood, and restoration of colon length in rats. Also, the DAI score was notably lower in the α-KG-treated (TNBS+AKG) group compared to the TNBS group (Fig. 2).

### 3.2 Effect of α-KG on TNBS induced colon histology

**Fig. 3:**
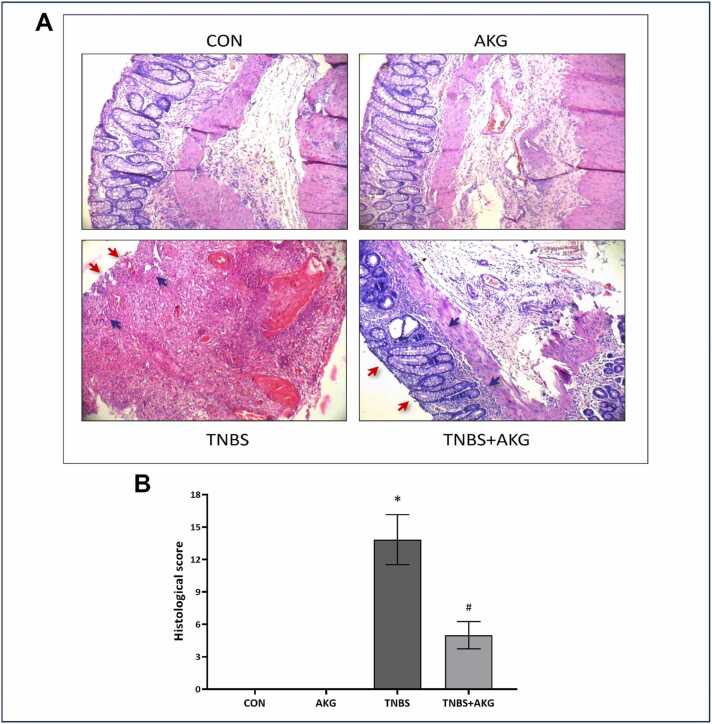
Effect of α-KG on colon histology. (A) Magnified photograph of colon histology from each group. Crypts are pointed by blue arrows and epithelial layer is pointed by red arrows in TNBS & TNBS+AKG. (B) Histological score of each experimental group. CON: animals were solely provided with a standard diet, AKG: experimental animals administered with α-KG dissolved in sterile water, TNBS: TNBS administered intrarectally for colitis induction, TNBS+AKG: α-KG administered orally 36 hours after TNBS administration and continued once every day for 5 days. The final results have been expressed as mean ± SEM of 3 independent experiments of each group. *P < 0.05 vs CON; #P < 0.05 vs TNBS.

Histopathological analysis revealed alteration & disruption in the epithelial and disarrayed architecture of colonic epithelial cells in TNBS group. However, treatment with α-KG resulted in improved histological appearance with restored crypt structure and healing epithelial surface (Fig. 3).

### 3.3 Effect of α-KG on cellular antioxidant machinery in induced colitis

**Fig. 4:**
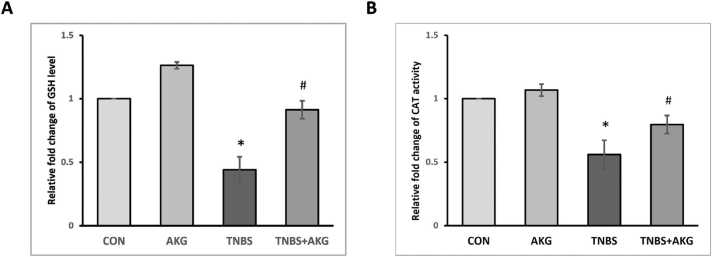
Effect of α-KG on cellular antioxidant machinery. Levels of (A) Relative fold change of GSH level in colon tissue, (B) Relative fold change of CAT activity in colon tissue. The final results have been expressed as mean ± SEM of 3 independent experiments of each group. CON: animals were solely provided with a standard diet, AKG: experimental animals administered with α-KG dissolved in sterile water, TNBS: TNBS administered intrarectally for colitis induction, TNBS+AKG: α-KG administered orally 36 hours after TNBS administration and continued once every day for 5 days. The final results have been expressed as mean ± SEM of 3 independent experiments of each group. *P < 0.05 vs CON; ^#^P < 0.05 vs TNBS.

Results obtained from this study showed decreased level of GSH and CAT activity in TNBS group, which was elevated after α-KG administration (Fig. 4).

### 3.4 Effect of α-KG on inflammation

**Fig. 5:**
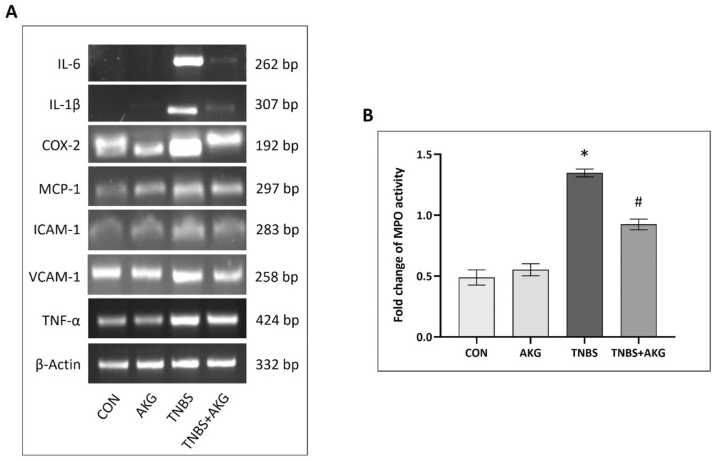
Effect of α-KG on inflammation. (A) mRNA expression levels of IL-6, IL-1β, COX-2, MCP-1, ICAM-1, VCAM-1 and TNF-α. (B) Colonic MPO activity in different experimental groups. CON: animals were solely provided with a standard diet, AKG: experimental animals administered with α-KG dissolved in sterile water, TNBS: TNBS administered intrarectally for colitis induction, TNBS+AKG: α-KG administered orally 36 hours after TNBS administration and continued once every day for 5 days. The final results have been expressed as mean ± SEM of 3 independent experiments of each group. *P < 0.05 vs CON; ^#^P < 0.05 vs TNBS.

With the aim to explore the effect of α-KG on inflammation, mRNA expression of pro-inflammatory cytokines and chemokines were examined. mRNA expression of TNF-α, IL-1β, IL-6, COX-2, ICAM-1 & VCAM-1 and MCP-1 were seen upregulated following TNBS administration. Treatment with α-KG reduced the mRNA expression of these pro-inflammatory cytokines. MPO activity in TNBS group was seen elevated which was reduced after treatment with α-KG (Fig. 5).

### 3.5 Effect of α-KG on endoplasmic reticulum stress induced apoptosis

**Fig. 6:**
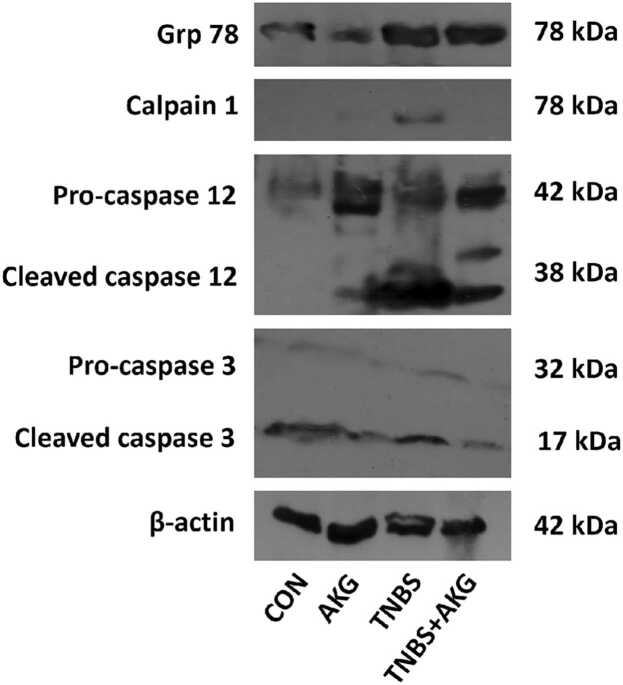
Effect of α-KG on endoplasmic reticulum stress induced apoptosis. Immunoblot analysis of GRP78, calpain 1, caspase 12 and caspase 3 protein expression in colon tissue. CON: animals were solely provided with a standard diet, AKG: experimental animals administered with α-KG dissolved in sterile water, TNBS: TNBS administered intrarectally for colitis induction, TNBS+AKG: α-KG administered orally 36 hours after TNBS administration and continued once every day for 5 days.

In this study, we observed upregulation in levels of GRP78, calpain 1 and caspase 12 which indicated initiation of ER stress upon TNBS administration. However, α-KG treatment significantly reduced the elevated levels of such molecules. An elevation in caspase 3 expression was also observed in colon of TNBS group while treatment with α-KG lowered the expression of this apoptotic molecule (Fig. 6 and Supplementary Fig. 1).

### 3.6 Molecular Docking Simulations analysis

**Fig. 7:**
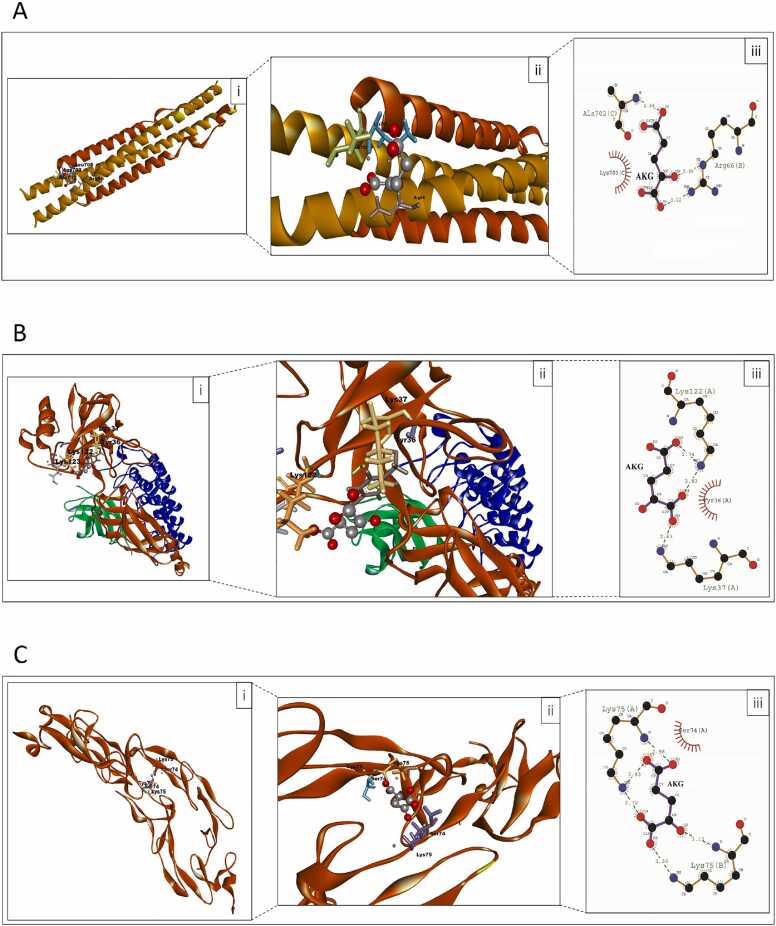
The receptor protein-ligand interactions of A. IKK complex, B. NF-κB complex, C. TNFR-1 complex with α-KG. Three-dimensional structures showing α-KG binding pocket of IKK complex, NF-κB complex and TNFR-1 complex respectively [A. i. & ii., B. i. & ii., C. i. & ii.]. Ligplots [A. iii., B. iii. and C. iii.] were generated using LigPlot+ which gives an overview about two-dimensional representation of hydrogen bond and hydrophobic interactions of ligand-protein complexes.

The modes of the binding interactions between the receptor and the ligand were calculated using the DS2.5 platform. It was observed that ligand interacts maximally with NF-κB followed by TNFR-1 and IKK as reflected by their binding free energy values (Table 4). The patterns of the binding interactions were depicted in (Table 5) (Fig. 7).

**Table 4:** Autodock 4.2 binding free energy values of IKK, NF-κB and TNFR1 protein with the ligand α-KG.

**Protein**	**PDB ID**	**Chain information**	**Estimated free energy of binding (kcal/mol)**	**Estimated Inhibition constant**
IKK	3BRV	A, C chain: chain Inhibitor of NF-κB kinase subunit beta (red), B, D chain: NF-κB essential modulator (golden)	−3.8	1630 µM
NF-κB	1NFI	A chain: NF-κB p65 (red), B chain: NF-κB p50 (green), F chain: IκBα (blue)	−5.28	134.72 µM
TNFR	1EXT	A, B chain: TNFR (red)	−4.73	342.42 µM

**Table 5:** Interacting residues of IKK, NF-κB and TNFR1 protein with the ligand α-KG.

**Name of the receptor protein**	**Interacting amino acid residues of the receptor protein**
IKK complex	Arg66 (B), Ala702 (C), Lys703 (C), Leu708 (C)
NF-κB	Tyr36 (A), Lys37 (A), Lys122 (A), Lys123 (A)
TNFR1	Cys73 (A), Ser74 (A), Lys75 (A), Ser74 (B), Lys75 (B)

## 4 Discussion

In this study, we investigated the ameliorative effects of α-KG in colitis induced by TNBS. TNBS induces colitis in the experimental rats by compromising the intestinal epithelial barrier and activating a Th1/Th17-driven immune response. Ethanol facilitates TNBS penetration into the colonic mucosa, where TNBS acts as a hapten and binds to proteins, triggering an amplified inflammatory reaction [42], [43]. The onset of colitis was verified by symptoms of frequent soft stool with blood and decreased appetite, which started to manifest at around 24 hours of TNBS administration, followed by gradual weight loss. A reduction in colon Length was observed in diseased rats. Histopathological examination of colon tissue revealed distortion of the epithelial and cryptic structures of intestinal epithelium. Our research demonstrated that treatment with α-KG improved these alterations.

One of the pathological phenomena linked with colitis is oxidative stress [44]. Prolonged accretion of ROS leads to excessive production of superoxide radicals. The enzyme SOD neutralizes ROS by converting them to H_2_O_2_. Next, the enzyme CAT converts H_2_O_2_ into H_2_O and O_2_. Additionally, GSH mitigates oxidative stress by converting H_2_O_2_ into water and oxygen, facilitated by the enzyme glutathione peroxidase [45], [46]. After TNBS administration, perturbation of redox status in the colonic tissue was noticed, as indicated by decrease in GSH level and CAT activity. Treatment with α-KG increased the level of GSH as well as elevated CAT activity. Our results indicated that α-KG mitigated colitis by reducing oxidative stress.

Evidences suggest that pro-inflammatory cytokines are actively involved in the initiation of inflammatory processes in colitis [47], [48]. Coupled with ROS, pro-inflammatory cytokines increase the expression of chemokines and cell adhesion molecules which promote employment of more inflammatory cells thereby amplifying inflammatory response. Our study revealed the protective role of α-KG against the inflammatory process by down-regulating mRNA expression of pro-inflammatory cytokines (TNF-α, IL-1β and IL-6), cell adhesion molecules (ICAM-1 and VCAM-1) and chemoattractant protein (MCP-1). These molecules were seen upregulated after induction of colitis. Increased expression of COX-2 was also noted in colitis, suggestive of inflammation via production of prostaglandin, which was reduced upon treatment with α-KG. Neutrophil infiltration in colonic tissue in colitis induced rat was indicated by elevated MPO activity and it was found to be reduced upon α-KG treatment in our study.

Excessive accumulation of ROS causes oxidative stress in ER thereby impairing the mechanism of proper protein folding [49]. Accumulation of unfolded proteins in the ER augments UPR through activation of GRP78. This GRP78 is a chaperone protein which helps to resolve the stress generated by unfolded proteins via activation of several molecules in the pathway [50]. Our study investigated the effect of activation of GRP78 following colitis induction. In this study, we observed that protein expression of GRP78 was upregulated in colitis indicating ER stress, while treatment with α-KG decreased that expression, suggesting a reduction in that stress.

Persistent oxidative stress, inflammation and ER stress lead to disturbance in calcium homeostasis. Release of calcium ions into the cytosol activates calpain and this activated calpain converts pro-caspase 12 to caspase 12. The activated form of caspase 12 induces cell death [51], [52]. Our results suggested that treatment with α-KG reduced activation of calpain-1 and caspase 12. Inflammation and oxidative stress lead to depolarization of mitochondrial membrane with upregulation of caspase 3 level resulting in apoptosis [30], [53], [54], [55], [56]. According to our observations, the level of caspase 3 was found to be elevated in rats with colitis, but administration of α-KG reduced the increased level of caspase 3. This indicated a decrease in apoptosis after α-KG treatment.

Molecular docking simulations were utilized for extended analysis of the bonding affinity between the receptor proteins and α-KG. The values of binding free energy of the receptor ligand complexes were calculated. It was noted that α-KG has the highest binding affinity for NF-κB complex than IKK and TNF-R1.

**Fig. 8:**
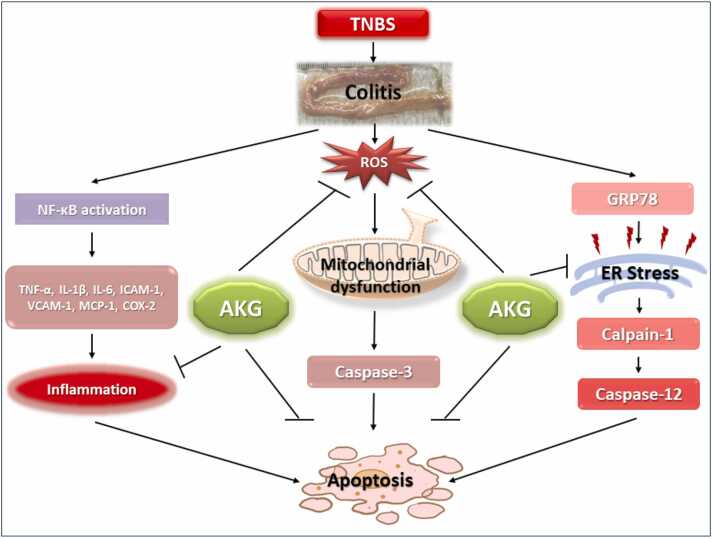
Schematic representation of the molecular mechanistic details of the protective effects of α-KG against colitis induced pathophysiology.

In conclusion, our results suggest that α-KG possesses the ability to mitigate colitis by modulating oxidative stress, inflammatory cytokines, ER stress and apoptosis. These observations may motivate further investigation to identify the potency of α-KG as a supplementary therapeutic agent in the treatment of colitis (Fig. 8).

## Funding

The infrastructural support from the DBT-funded Bioinformatics Infrastructure Facility Centre (Project Sanction no: BT/PR40162/BTIS/137/48/2022, dated 31.10.2022) and National Network Project (Project Sanction no: BT/PR40192/BTIS/137/69/2023, dated 19.12.2023), both of which have been sanctioned to Prof. Angshuman Bagchi, were utilized in insilico part of the work.

## CRediT authorship contribution statement

**Parames Sil:** Writing – review & editing, Writing – original draft, Visualization, Validation, Supervision, Resources, Project administration, Investigation, Funding acquisition, Conceptualization. **Sima Biswas:** Writing – review & editing, Writing – original draft, Visualization, Validation, Software, Methodology, Investigation, Formal analysis, Data curation, Conceptualization. **Angshuman Bagchi:** Writing – review & editing, Visualization, Validation, Supervision, Software, Resources, Project administration, Investigation, Conceptualization. **Sharmistha Banerjee:** Writing – review & editing, Writing – original draft, Visualization, Validation, Software, Methodology, Investigation, Formal analysis, Data curation, Conceptualization. **Sumit Ghosh:** Writing – review & editing, Writing – original draft, Visualization, Validation, Software, Methodology, Investigation, Formal analysis, Data curation, Conceptualization. **Ankita Mandal:** Writing – review & editing, Writing – original draft, Visualization, Validation, Software, Methodology, Investigation, Formal analysis, Data curation, Conceptualization.

## Declaration of Competing Interest

The authors declare that they have no known competing financial interests or personal relationships that could have appeared to influence the work reported in this paper.

## Data Availability

Data will be made available on request.
