# Supplementary material for: α-ketoglutarate ameliorates colitis through modulation of inflammation, ER stress, and apoptosis
Source: Toxicol Rep. 2025 Jan 6;14:101897. doi: 10.1016/j.toxrep.2025.101897 (PMC11780160; doi:10.1016/j.toxrep.2025.101897)
Supplement: Supplementary file 1 — Supplementary material [file mmc1.doc]

**α-ketoglutarate ameliorates colitis through modulation of inflammation, ER stress and apoptosis**

**Ankita Mandal^1*^, Sharmistha Banerjee^1*^, Sumit Ghosh^1^, Sima Biswas^2^, Angshuman Bagchi^2^ & Parames C. Sil^1**^**

***^1^Division of Molecular Medicine, Bose Institute, P-1/12, CIT Scheme VII M, Kolkata-700054, West Bengal, India.***

***^2^Department of Biochemistry and Biophysics, University of Kalyani, Kalyani-741235, Nadia, West Bengal, India.***

**^*^*AM & SB both contributed equally.***

**^**^ Address for correspondence**

Parames C. Sil

Professor, Division of Molecular Medicine

Bose Institute

P-1/12, CIT Scheme VII M

Kolkata-700054

West Bengal, India

E-mail: parames@jcbose.ac.in, parames_95@yahoo.co.in


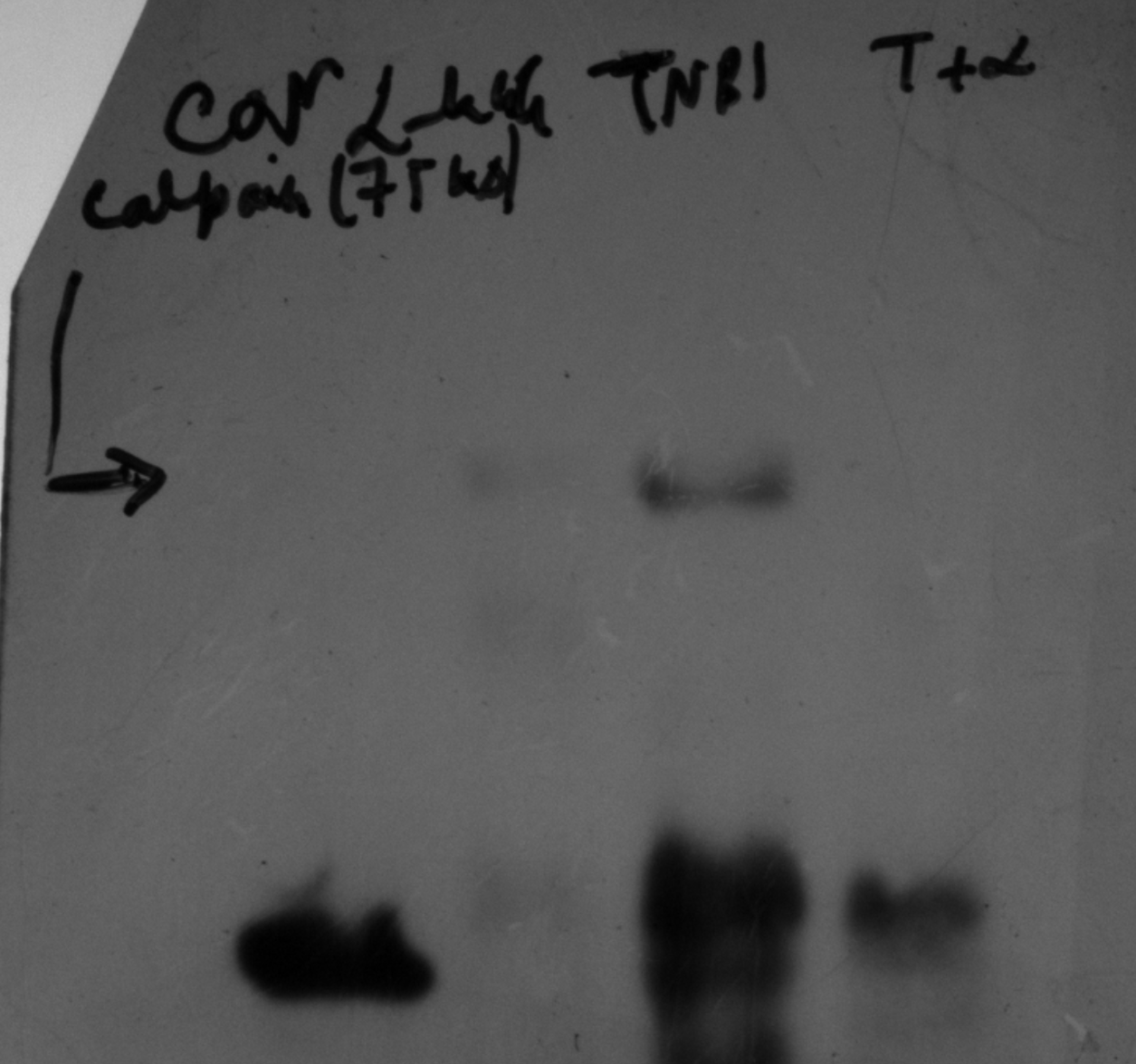


**Calpain 1 (78 kDa)**


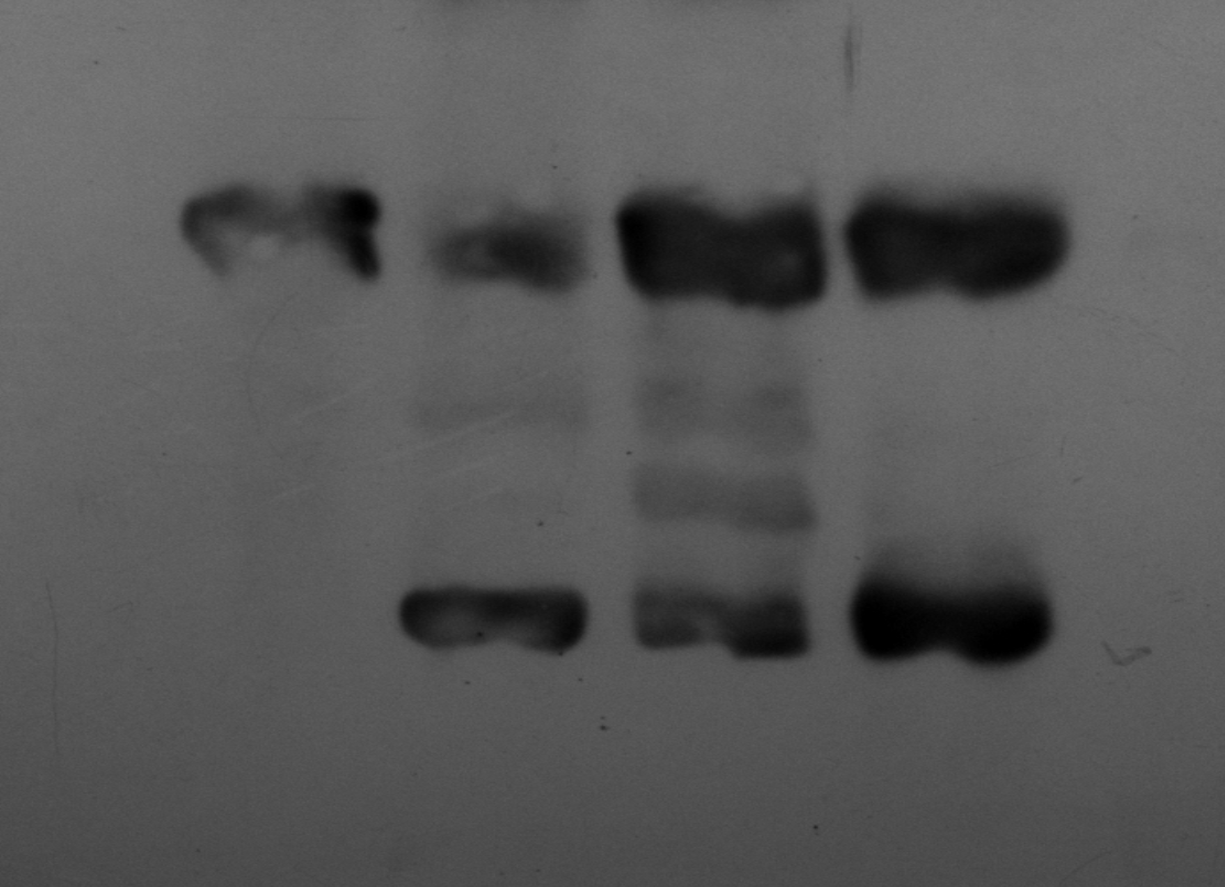


**Grp78 (78 kDa)**


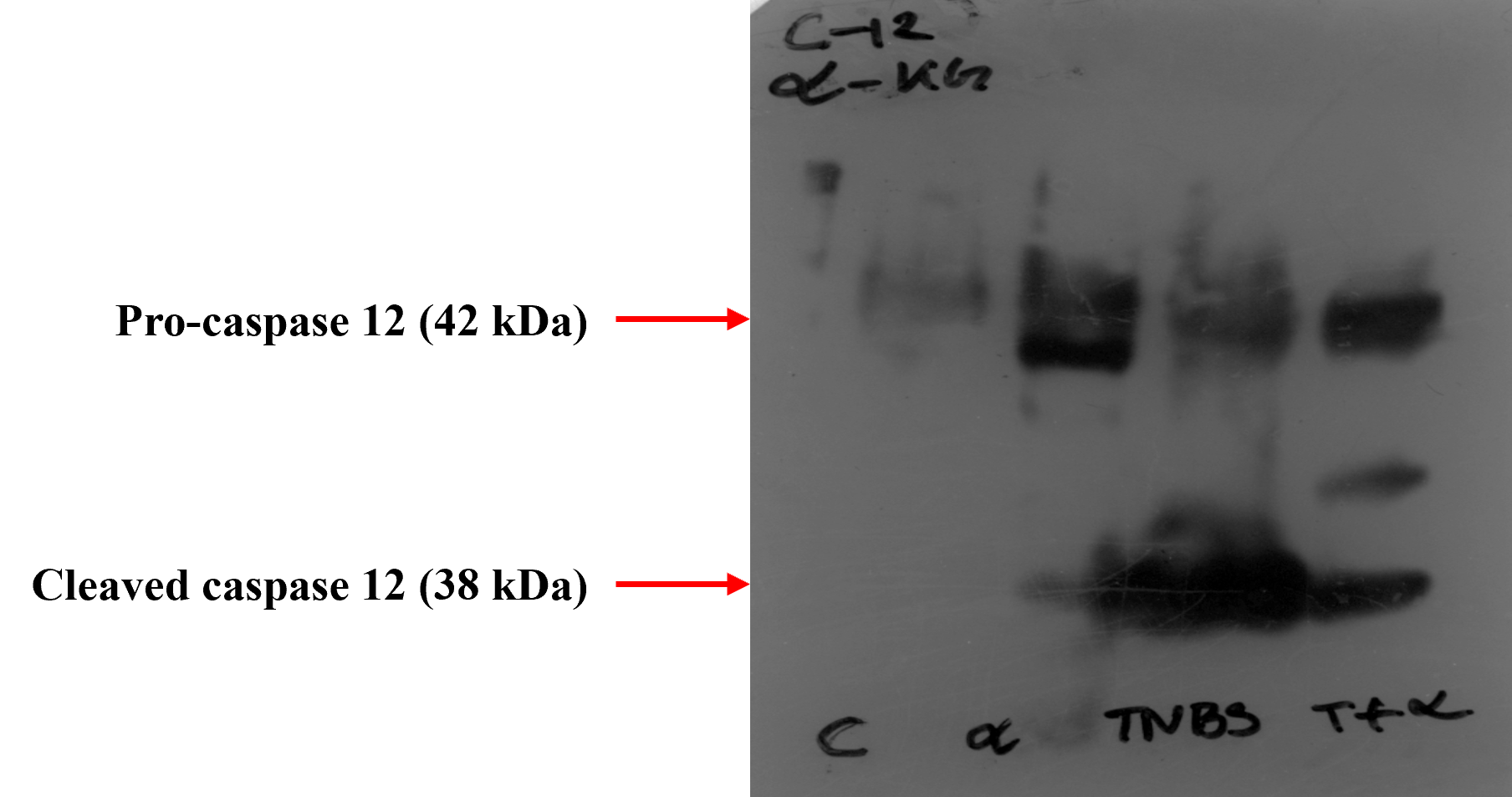


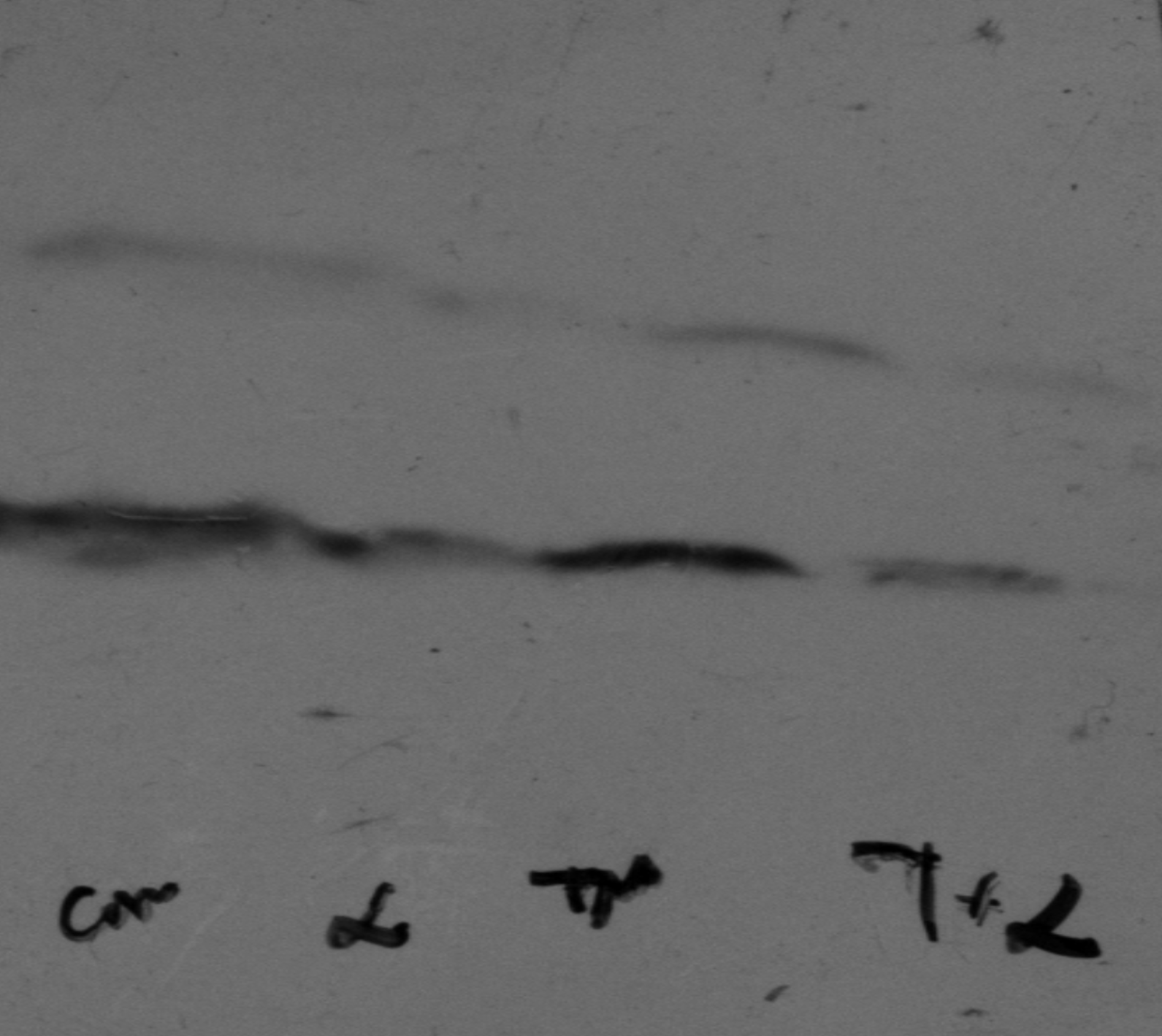


**Pro-caspase 3 (32 kDa)**

**Cleaved caspase 3 (17 kDa)**


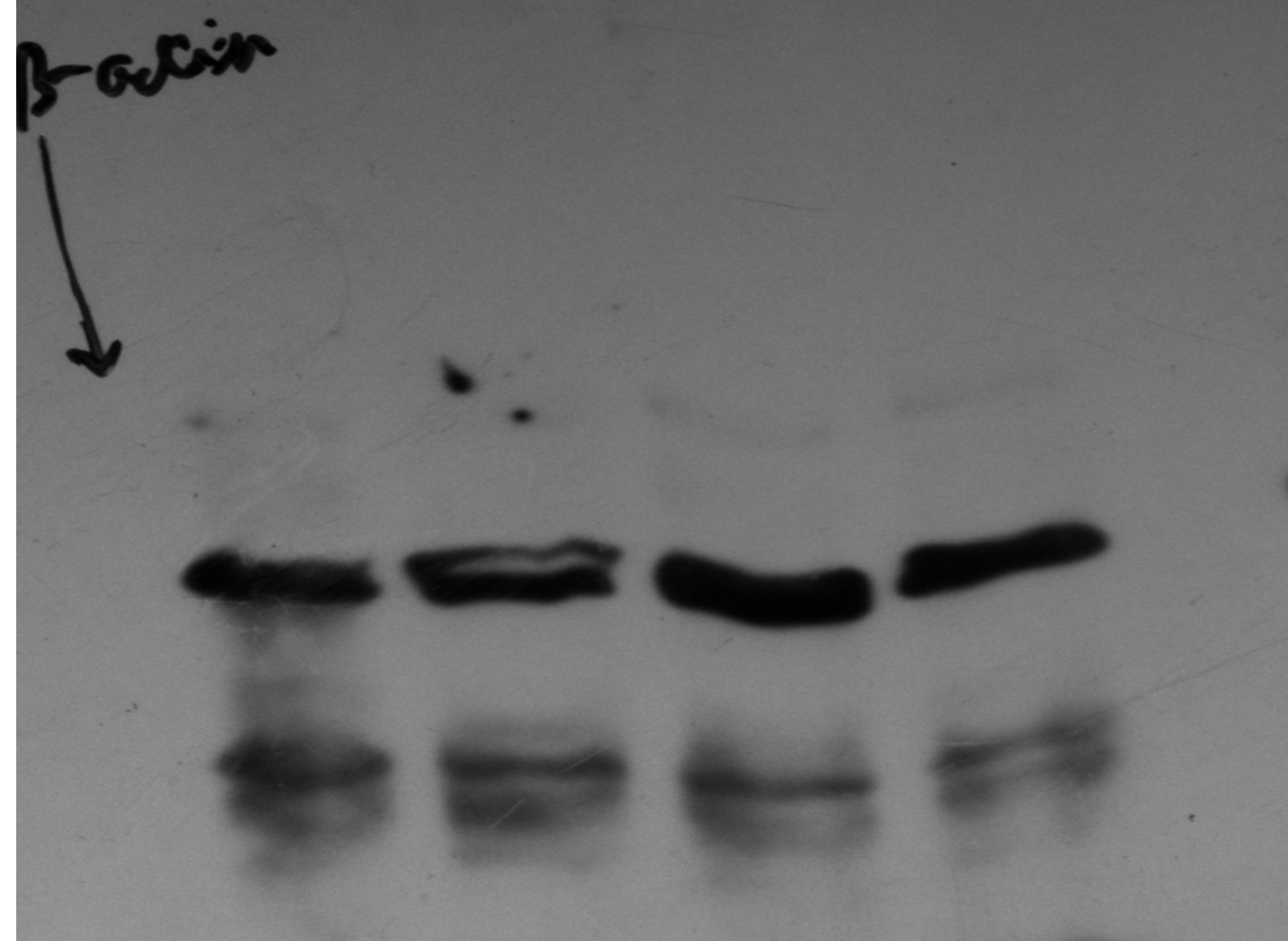


**β actin (42kDa)**

**Supplementary figure 1. Full uncropped blots of Figure 6.** Immunoblot analysis of GRP78, calpain 1, caspase 12, caspase 3 and β actin protein expression in colon tissue.
